# Supplementary material for: WormSpot: a machine learning-powered viability scoring platform in C. elegans for Candida pathogenicity studies
Source: Microbiol Spectr. 2026 May 12;14(6):e00252-26. doi: 10.1128/spectrum.00252-26 (PMC13228051; doi:10.1128/spectrum.00252-26)
Supplement: Supplemental figures — Fig. S1 to S3. [file spectrum.00252-26-s0001.docx]

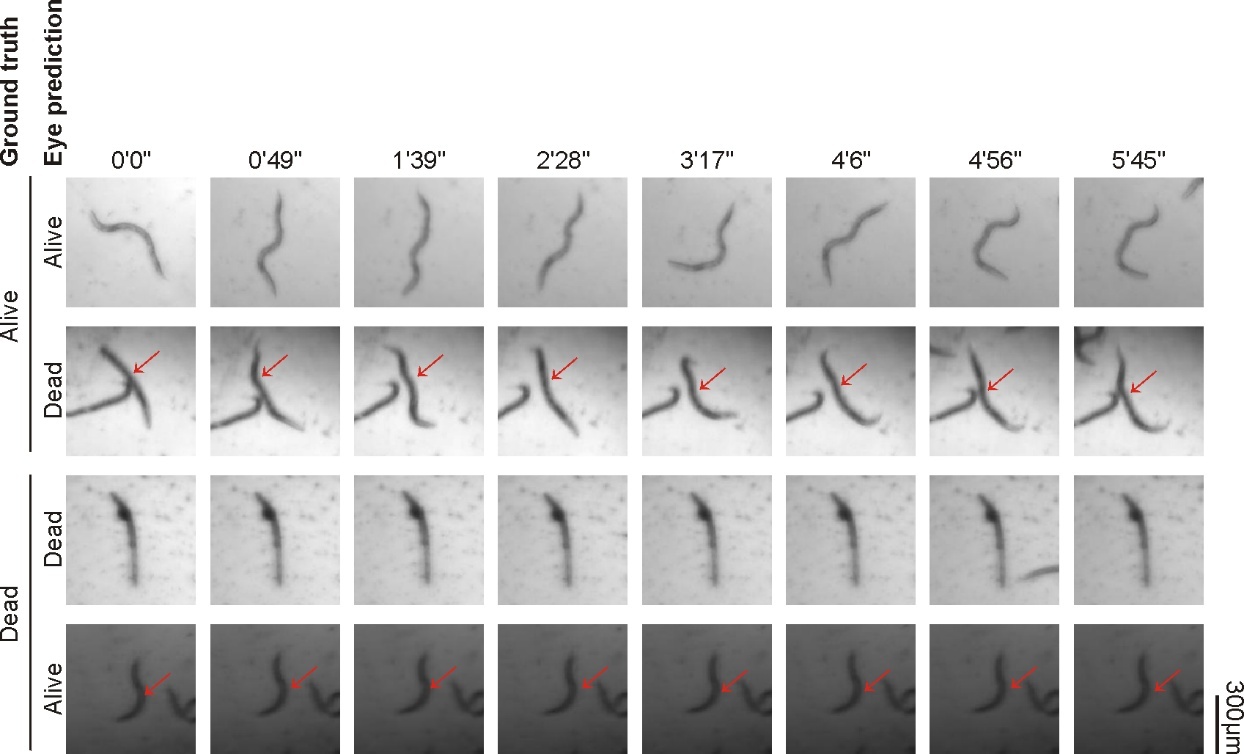


**Figure S1. Video verification of worm viability.**

Example video frames showing correct and incorrect eye predictions of 'Alive' and 'Dead' worms. Red arrows indicate the misclassified worms. Scale bar, 300 μm.


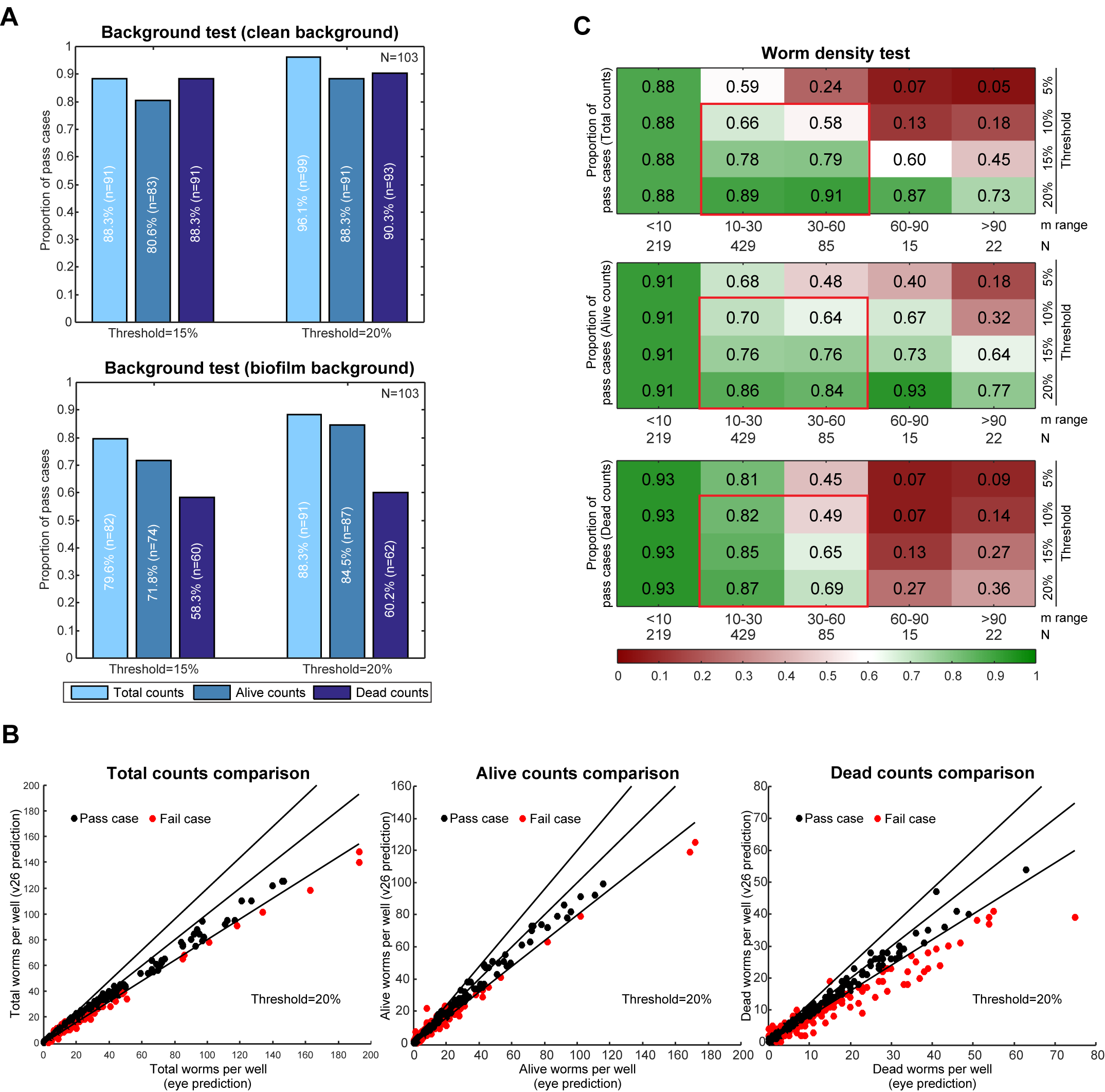


**Figure S2. Assessing WormSpot effectiveness for well-based predictions.**

(A) Bar plots separately showing the fraction of pass cases for clean and biofilm-dense images (N = 103 images each) in the background test at 15% and 20% tolerance thresholds for the indicated counts. N, total number of images; n, number of pass cases. (B) Graphs comparing WormSpot predictions (Y-axis) with eye predictions (X-axis) for ‘Total’, ‘Alive’, and ‘Dead’ worm counts in the worm density test. The diagonal line represents exact matches, while the flanking lines indicate 20% threshold boundaries. Dots within the threshold are shown in black; those outside are shown in red. (C) Heatmap showing fractions of pass cases for indicated counts in the worm density test across five specified m ranges under four tolerance thresholds (5%, 10%, 15%, 20%). m, eye-annotated worm count per well; N, image number per m range.

**
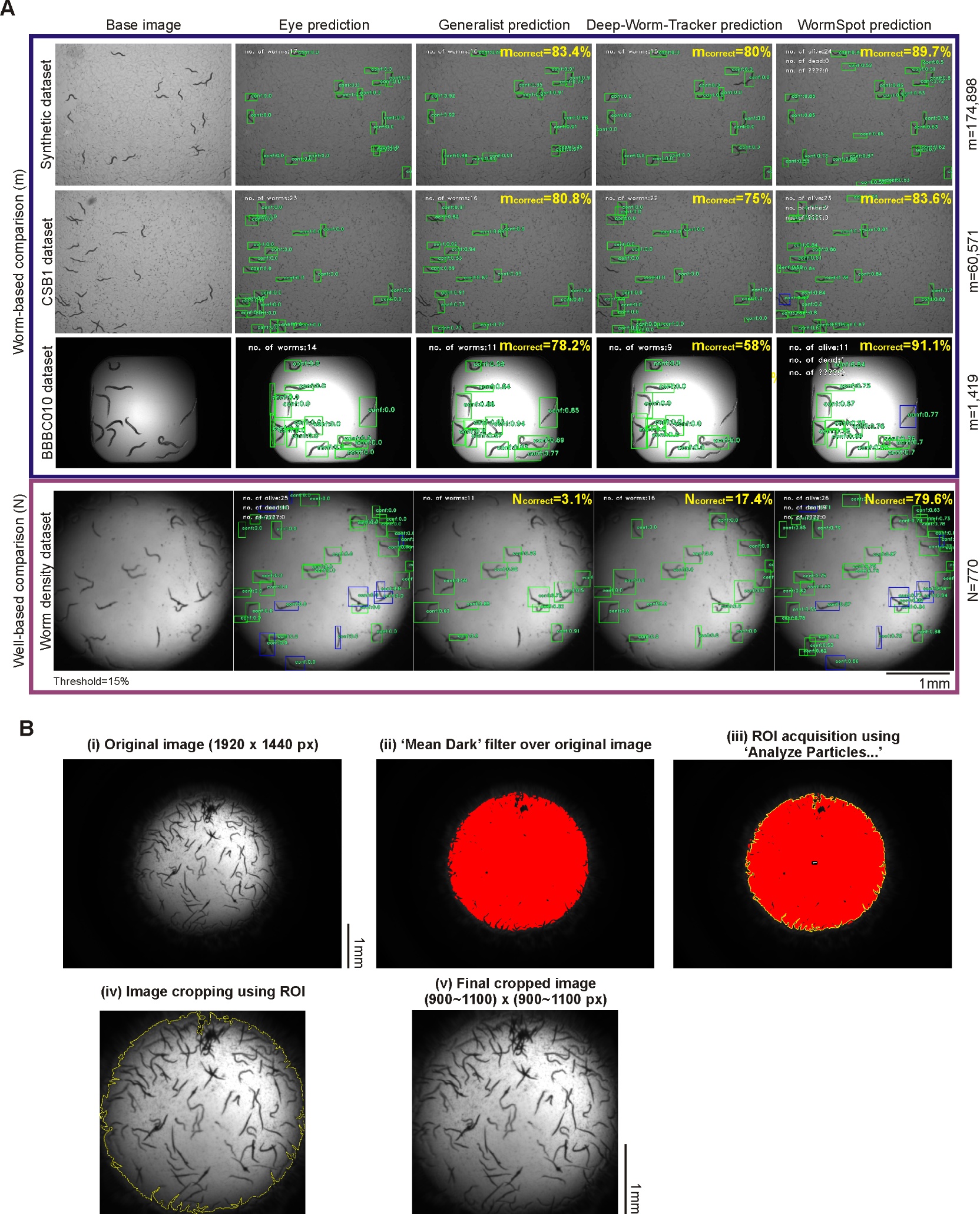
**

**Figure S3. Comparison of worm detection performance between WormSpot, Generalist, and Deep-Worm-Tracker.**

(A) Comparison of worm-based (blue frame) and well-based (purple frame) worm detection performance across WormSpot, Generalist and Deep-Worm-Tracker. Worm-based comparisons used indicated public image datasets, while well-based comparisons used our worm density test dataset at a 15% tolerance threshold. Example images and their corresponding eye annotations and model predictions are shown. Eye-annotated total worm counts (m) for each dataset or well/image counts (N) for the worm density test dataset are listed on the right. We define m_correct_ as the percentage of worms correctly detected and N_correct_ as the percentage of ‘pass’ cases at the 15% tolerance threshold. (B) Example images showing ImageJ processing steps for generating the final cropped images used in training and testing. Scale bar, 1 mm.
